# Quantitative Evaluation of Hypomimia in Parkinson’s Disease: A Face Tracking Approach

**DOI:** 10.3390/s22041358

**Published:** 2022-02-10

**Authors:** Elena Pegolo, Daniele Volpe, Alberto Cucca, Lucia Ricciardi, Zimi Sawacha

**Affiliations:** 1 Department of Information Engineering, University of Padova, 35131 Padova, Italy; elena.pegolo@phd.unipd.it; 2 Fresco Parkinson Center, Villa Margherita, Santo Stefano Riabilitazione, 36057 Arcugnano, Italy; dott.volpe@libero.it (D.V.); alberto.cucca@casadicuravillamargherita.it (A.C.); 3 Department of Life Sciences, University of Trieste, 34127 Trieste, Italy; 4 Department of Neurology, School of Medicine, New York University, New York, NY 10016, USA; 5 Neurosciences Research Centre, Molecular and Clinical Sciences Research Institute, St. George’s University of London, London SW17 0RE, UK; lucia.ricciardi2@gmail.com; 6 Medical Research Council Brain Network Dynamics Unit, Nuffield Department of Clinical Neurosciences, University of Oxford, Oxford OX3 9DU, UK; 7 Department of Medicine, University of Padova, 35131 Padova, Italy

**Keywords:** Parkinson’s disease, facial expression, Facial Action Coding System, feature tracking, emotions, classification, face, hypomimia

## Abstract

Parkinson’s disease (PD) is a neurological disorder that mainly affects the motor system. Among other symptoms, hypomimia is considered one of the clinical hallmarks of the disease. Despite its great impact on patients’ quality of life, it remains still under-investigated. The aim of this work is to provide a quantitative index for hypomimia that can distinguish pathological and healthy subjects and that can be used in the classification of emotions. A face tracking algorithm was implemented based on the Facial Action Coding System. A new easy-to-interpret metric (face mobility index, *FMI*) was defined considering distances between pairs of geometric features and a classification based on this metric was proposed. Comparison was also provided between healthy controls and PD patients. Results of the study suggest that this index can quantify the degree of impairment in PD and can be used in the classification of emotions. Statistically significant differences were observed for all emotions when distances were taken into account, and for happiness and anger when *FMI* was considered. The best classification results were obtained with Random Forest and kNN according to the AUC metric.

## 1. Introduction

Parkinson’s disease (PD) is a neurodegenerative disorder characterized by motor symptoms such as tremor, rigidity, bradykinesia, and gait and balance problems. There is also a plethora of non-motor symptoms that are experienced by PD individuals and that have a strong impact on patients’ and their care-partners’ quality of life [1]. Emotional processing is impaired at different levels in PD [2] including facial expressivity and facial emotion recognition. Hypomimia/amimia is a term used to describe reduced facial expression in PD, which is one of the most typical features of the disease [3]. Despite being clinically well recognized, its significance, pathophysiology, and correlation with motor and non-motor symptoms is still poorly explored [4,5]. This is partially due to the scarcity of objective and validated measures of facial expression [6].

Face expressions are an important natural means of communicating, and have been the objective of several studies since the beginning of the 20th century [7] in healthy and different clinical populations. Hjortsjo [8] provided an anatomic description of muscular movements during facial expressions and their subdivision depending on the displayed emotions. Around the same period, other authors approached a subdivision of the meaning of the expressions by their inherent emotionality. This can be found in the work of Ekman and Friesen [9], who defined a precise small universal discretization of the six basic emotions according to Darwin [10] as follows: fear, anger, disgust, happiness, sadness, and surprise. Furthermore, the Facial Action Coding System (FACS) [9] was developed that describes facial expressions by means of action units (AUs). Of 44 defined FACS AUs, 30 AUs are anatomically related to the activation of specific facial muscles, and they can occur either individually or in combination. Through this encoding system, more than 7000 different AU combinations have been observed [9]. This system is still used in manifold fields and applications.

The analysis of facial expressions has advanced in many domains, such as face detection, tracking, pattern recognition, and image processing. In recent years, different algorithms and architectures have been proposed in Facial Expression Recognition (FER) systems. In order to extract relevant information for face and facial expression analysis they generally follow three main steps:

(a) Face landmark detection: Identification of landmarks is based on specific face features positions (i.e., eyes, mouth, nose, eyebrows, etc.). Usually, after landmarks have been detected, a step of normalization is performed by aligning each face to a local coordinate framework in order to reduce the large variation introduced by different faces and poses [11].
(b) Feature extraction: Feature construction and/or selection is usually based on the coordinates obtained from (a), and either an appearance or a geometric approach can be used. The former employs the texture of the skin and facial wrinkles, whereas the latter employs the shape, i.e., distances and angles of facial components [12].
(c) Classification: The last step concerns the classification of different emotions or expressions. Different methods are applied in the literature depending on the previous phases. The most-used classification algorithms in conventional FER approaches include Support Vector Machines, Adaboost, and Random Forest [13].

At present, algorithms for automatic facial analysis employing these kinds of methodologies are gaining increasing interest. The aims of these systems are facial comparison and/or recognition (e.g., OpenFace software [14]), in addition to the identification and classification of different emotions (e.g., EmoVu, FaceReader [15], FACET, and Affectiva Affdex [16]). Regards the latter objective, it is crucial to note that these algorithms usually adopt machine or deep learning (DL) techniques that exploit enormous databases of healthy subjects’ images. When using these methods to assess impairments in face mobility in a given pathology (e.g., PD, depression, obsessive-compulsive disorder [17]), the evaluation of the symptom is based on the measurement of the deviation of the acquired expressions from the corresponding ones in healthy individuals. Despite the growing interest in the application of FER algorithms to hypomimia, in particular to PD [4,18,19], there is still a paucity of work regarding the quantitative assessment of the degree of impairment in these individuals.

Emerging literature points towards the quantification of hypomimia as a potential marker for diagnosis and disease progression in PD, and some attempts in this area have been recently made. Bandini et al. [20] evaluated hypomimia in a cohort of PD subjects. They estimated a quantitative measure from the neutral expression in a subset of basic emotions (happiness, anger, disgust, and sadness), considering both the actuated and the imitated ones. Grammatikopoulou and colleagues [21] proposed an innovative evaluation of this symptom in PD based on images captured by smartphones. Two different indexes of hypomimia were developed without discriminating among different emotions. A review of automatic techniques for detecting emotions in PD was recently carried out by Sonawane and Sharma [22]; they investigated both machine and DL algorithms used in the classification of emotions in PD subjects with hypomimia. Moreover, they addressed the problem of expression quantification and related pending issues. In 2020, Gomez and colleagues [19] proposed a DL approach to model hypomimia in PD exploring different domains. The main issue they encountered when using such techniques was the lack of large databases of PD subjects’ videos and/or images to be exploited in this approach. In summary, the current state-of-the-art hypomimia evaluation proposes methodologies that aim, first, to distinguish PD and healthy control subjects, and second to develop quantitative metrics. The indexes available to date still have some limitations, such as the assessment of the symptom without considering the specific face muscles involved or the disregard of the basic emotions in the analysis [5]. The objective of the present study is to provide a quantitative measure of hypomimia that tries to overcome some of these limitations and is both able to differentiate between pathological and physiological states and classify the basic emotions.

In particular, the main contributions of this work are:

- the design of a new index based on facial features to quantify the degree of hypomimia in PD and link it to the different emotions;
- the definition of a stand-alone metric able to quantify the degree of hypomimia in each subject independently from the comparison with healthy subjects’ databases, thus enabling tracking of disease progression over the time;
- a spatial characterization in face regions strictly related to the movement of specific muscles, thus enabling targeting specific rehabilitation treatments.

## 2. Materials and Methods

### 2.1. Participants

A total of 50 PD subjects and 20 healthy control (HC) subjects were enrolled for the study. Power analysis for sample size estimation was applied [23] (*p* = 0.05, power = 80%, values from [24], Appendix A). People with idiopathic PD were recruited from the Department of Casa di Cura ‘‘Villa Margherita’’ in Vicenza, and healthy controls were recruited from hospital personnel. This study was approved by the local ethics committee (ARS_PD1/100-PROT). A written informed consent was obtained from all participants. Data from 3 healthy subjects were discarded from the analysis due to artifacts in the video sequences. Table 1 reports the demographic data of the participants. For PD individuals, data on disease duration and Unified Parkinson’s Disease Rating Scale (UPDRS) Part III in the ON medication status were collected.

#### Inclusion and Exclusion Criteria

Patients were eligible for inclusion if they were diagnosed with Parkinson’s disease according to UK Brain Bank criteria. The diagnosis was reviewed by a movement disorders neurologist. Exclusion criteria were: presence of clinically significant depression (according to Diagnostic and Statistical Manual of Mental Disorders-V (DSM-V) criteria and Beck’s depression inventory (BDI-II) score >17); presence of dementia (according to DSM-V criteria and MMSE score < 24); presence of deep brain stimulation surgery.

### 2.2. Pipeline

**Figure 1:**
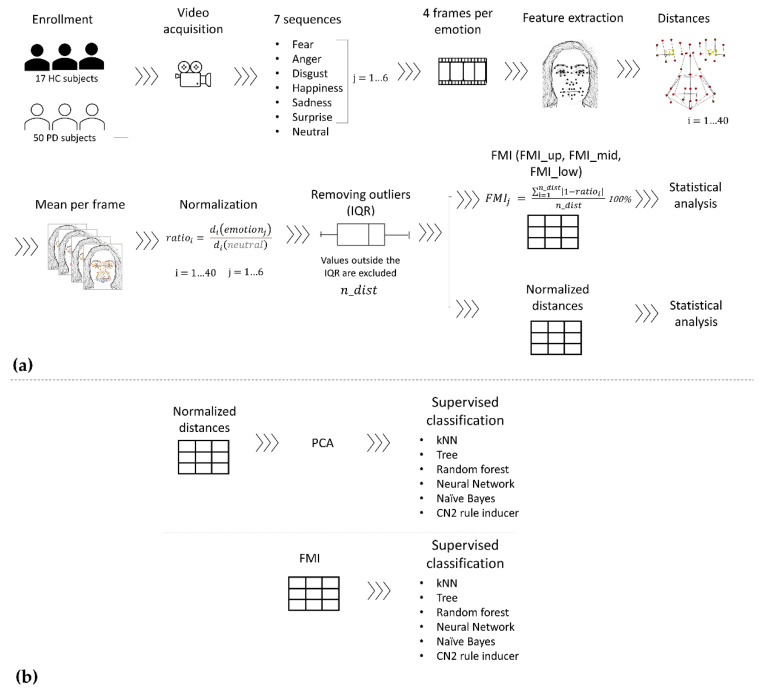
Data processing pipeline: (**a**) *FMI* definition from raw data to normalized distances and *FMI* calculation; (**b**) emotion classification with the normalized distances and *FMI* datasets. The outputs of pipeline (**a**), *FMI* and normalized distances datasets, are used as inputs for the classification pipeline (**b**).

A schematic representation of the processing pipeline is reported in Figure 1. Data acquisition, processing, and statistics are described in Section 2.2.1, Section 2.2.2 and Section 2.2.3 respectively. Data were imported into MATLAB (R2017a) and custom code was developed to perform the analysis. Moreover, unsupervised classification was implemented in Orange data mining toolbox [25], as described in Section 2.2.4.

#### 2.2.1. Data Acquisition

Frontal face videos of the participants were recorded while they were instructed by the researcher to perform, in random order, the six basic facial emotions: anger, disgust, fear, happiness, sadness, and surprise. The neutral face expression was also acquired either at the beginning or at the end of the video session while the participant was invited to remain silent and look at the video camera while resting. Subjects were comfortably seated in front of a commercial camera (GoPro Hero 3, 1920 × 1080 pixels, 30 fps) placed at eye level. A neutral background was located behind them [5].

#### 2.2.2. Data Processing

**Figure 2:**
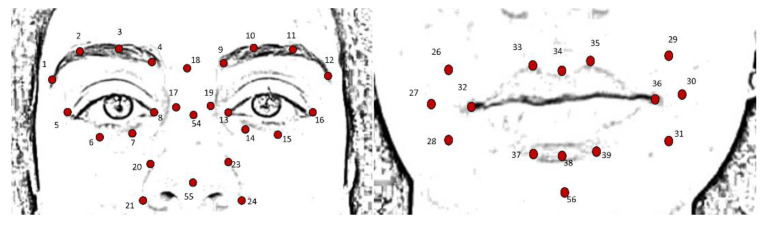
Landmark description: from 1 to 16 features describing the eyes, from 17 to 24, 54 and 55 describing the nose, from 25 to 39 and 56 describing mouth and cheeks.

**Figure 3:**
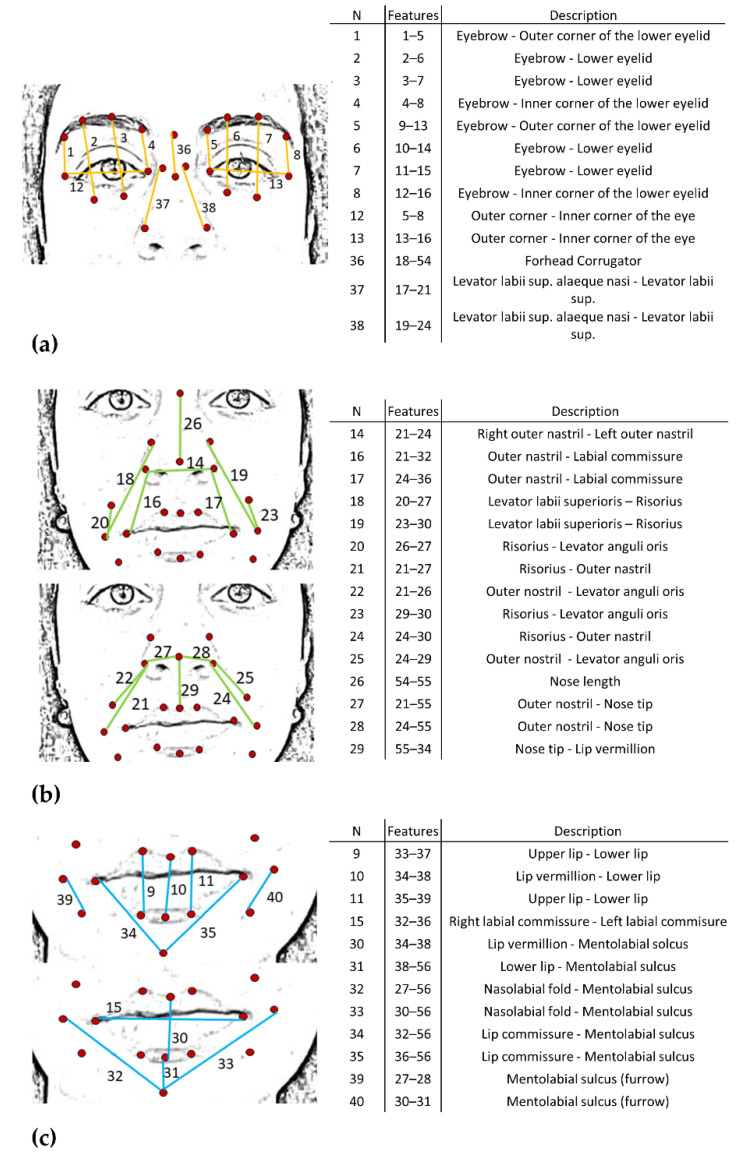
Description of the face distances. *N* = number of distances. Features = Pairs of features where the corresponding distance is calculated. Description = muscles related to the specific distance. (**a**) Upper face; (**b**) middle face; (**c**) lower face.

For each of the six emotions and the neutral expression, four frames were extracted from the acquired videos; these were selected as the frames immediately following the instruction given by the clinician. Based on the FACS encoding system, a set of facial landmarks was defined. This corresponds to forty points in the 2D space-image; Figure 2 describes the different landmarks. Following Cootes et al. [26], 3 types of facial feature points were adopted: points labeling parts of the face with application-dependent significance, such as the eyebrows and the lip contour (see Figure 2, feature numbers 1, 2, 3, 4 and 33, 34, 35); points labeling application-independent elements, such as curvature extrema (the highest point along the bridge of the nose, see feature numbers 18 on Figure 2); and points interpolated from the previous two types, such as feature numbers 19 and 23 (Figure 2). Each point was tracked with TrackOnField (BBSoF S.r.l. [27]). From the coordinates of these landmarks, forty Euclidean distances were computed (Figure 3) per frame.

Each obtained value was then averaged over the extracted frames, obtaining a single value per each distance. Then, each distance was normalized to the corresponding value in the neutral expression (Equation (1)).

(1) $$ratio_{i}=\frac{d_{i}\left(emotion_{j}\right)}{d_{i}\left(neutral\right)}\;\;\;\;i=1\ldots 40,\;\;\;j=1\ldots 6\;$$

Values outside the interquartile range were excluded from the analysis. Lastly, a total *FMI* was defined and calculated as follows:

(2) $$FMI_{j}=\frac{\sum_{i=1}^{n\_dist}\left|1-ratio_{i}\right|\cdot 100\%\;}{n\_dist}\;\;\;\;j=1\ldots 6\;$$

For each emotion (*j* = 1...6), the *FMI* was determined as the summation of the percentage deviation from the neutral expression ($\left|1-ratio_{i}\right|\cdot 100\%$) of all the distances; the *FMI* was then normalized to the number of available distances (*n_dist*). Overall, *FMI* represented an intuitive description of the mobility of face muscles in the different emotions with respect to the neutral expression.

Moreover, three indexes per face region were computed. The same formula as before was applied (Equation (2)) but distances were grouped according to Figure 3a–c. A space characterization was performed in the upper (*FMI_up*), middle (*FMI_mid*), and lower (*FMI_low*) parts of the face, respectively.

Finally, a further *FMI* was computed by considering only the statistically significant distances for each emotion (Appendix B).

#### 2.2.3. Statistics

Statistical analysis was performed in order to compare, first, the normalized distances, and then the different *FMIs*. Non-parametric tests were applied to the two cohorts of subjects and to each emotion. The Kruskal–Wallis test (*p* < 0.05) was implemented to compare the normalized distances (ratio in Equation (1)). The Wilcoxon rank sum test (*p* < 0.05) was used to compare the different *FMIs* (*FMI*, *FMI_up*, *FMI_mid*, *FMI_low*) between healthy and PD individuals.

Finally, a correlation analysis was performed between *FMIs* and values of UPDRS III, age, disease duration, and gender per each emotion in the PD cohort of subjects only. Pearson correlation coefficients (r) were computed for all the quantities, except for gender, which, being a binomial variable, required the use of the Point-biserial correlation coefficient (r_PB_) [28].

#### 2.2.4. Supervised Classification

Different supervised classification algorithms were applied. The following models were evaluated: k-Nearest Neighbors (kNN), Tree, Random Forest, Neural Network, Naïve Bayes, and CN2 rule inducer. Algorithms were applied to both normalized distances and the *FMI* of the two cohorts with the aim to discriminate the different emotions. The distances dataset only was preprocessed with a principal component analysis (PCA, 10 components, 81% explained variance) due to the presence of a high correlation among the data. Given the reduced dimension of the training datasets, test phases of the classification were performed with a leave-one-out cross validation in both datasets. In order to evaluate the best classification technique, the following standard performance metrics were calculated: area under the curve (AUC), F1 score, precision, and recall [29].

## 3. Results

### 3.1. Results of the Statistical Analysis of Distances and FMIs

**Figure 4:**
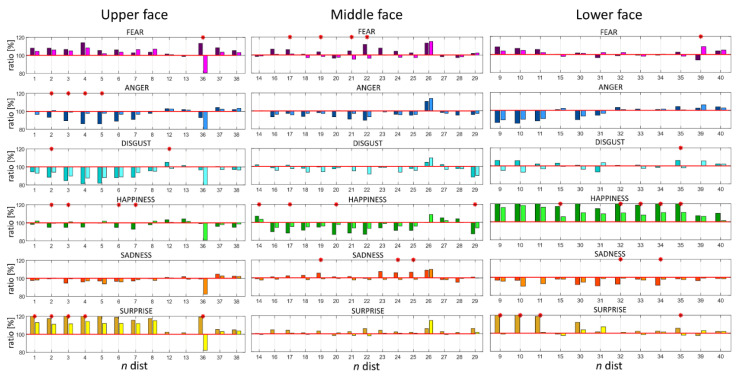
Values of normalized distances per each emotion in the three face regions: upper, middle, and lower. The x-axis represents the distance number, y-axis the values of ratios expressed in % from the neutral expression. Darker colors correspond to the HC population whereas lighter ones to PD. Red * highlights statistically significant differences at the 0.05 confidence level.

In reference to Figure 3 (column N) each normalized distance (Equation (1)) was characterized by a number. Figure 4 reports the normalized distances (ratio) per each emotion in the three face regions: upper, middle, and lower. Values greater than 100% represented an increase from the neutral expression in the specific distance and, conversely, while considering values lower than 100%. Therefore, the closer the distance to 100% the less the variation from the neutral expression. When comparing the two cohorts of subjects, statistically significant differences (*p* < 0.05) between corresponding distances were highlighted per each emotion (Figure 4 and Table 2). Results of the analysis for the *FMI* computed by considering only the distances reported in Table 2 can be found in Appendix B.

**Figure 5:**
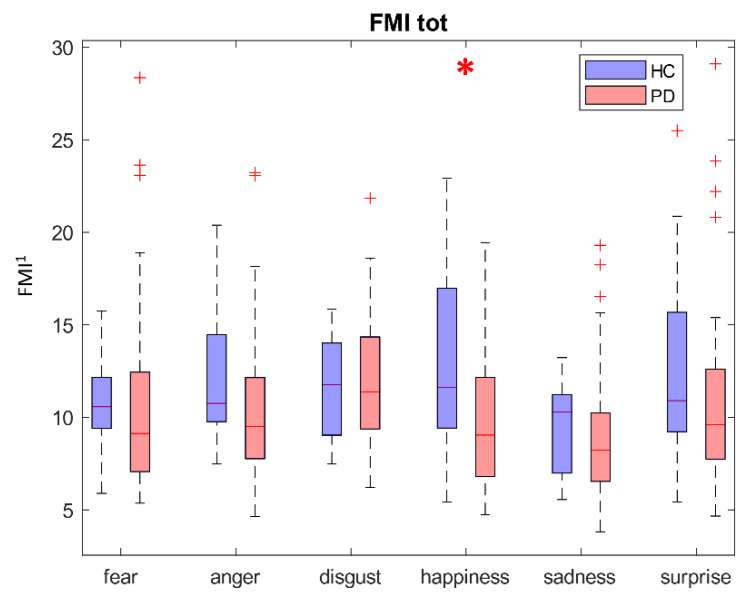
Boxplot of the total *FMI* per emotion. The x-axis represents the different emotions; the y-axis reports the value of *FMI*. HC and PD subjects are described in blue and red, respectively. Greater values of *FMI* represent greater deviation from the neutral expression. Red * highlights statistically significant differences at the 0.05 confidence level. *FMI*^1^ is dimensionless.

When combining all the distances in the *FMI*, the comparison between the two populations of subjects (see Figure 5) revealed statistically significant differences only in the happiness emotion (*p* < 0.05), even though HC subjects displayed a higher absolute value for almost all the emotions.

**Figure 6:**
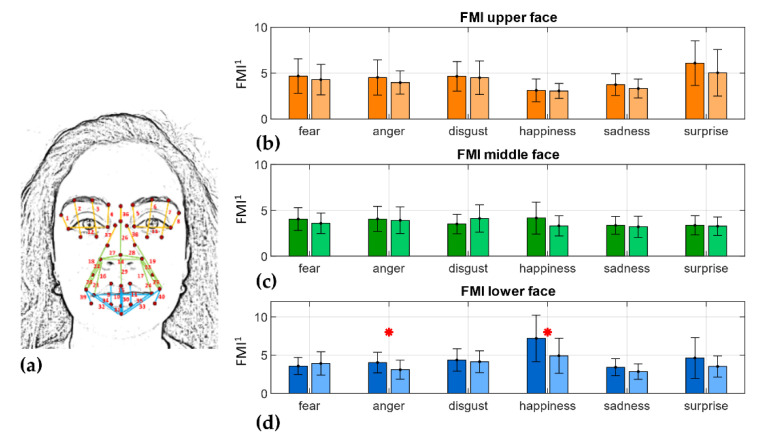
*FMI* per face region. (**a**) Distances grouped by face region; orange: upper face, green: middle face, blue: lower face; (**b**) *FMI_up*; (**c**) *FMI_mid*; (**d**) *FMI_low*. Darker colors refer to HC subjects, lighter colors to the PD cohort of subjects. Bar plot of the *FMI* values are reported as mean ± SD per cohort. Red * highlights statistically significant differences at the 0.05 confidence level. *FMI*^1^ is dimensionless.

When considering the *FMI* associated with the three face regions (Figure 6d), it can be noted that the lower (Figure 6c) part index was the only one that displayed statistically significant differences between the two populations of subjects in both anger and happiness emotions (*p* < 0.05).

In Table 3, the correlation coefficients between *FMIs* and clinical and demographic variables (UPDRS III, duration of the disease, age, gender) per each emotion are reported. The analysis was performed on the PD cohort of subjects only and values of *FMI* were employed. No statistically significant correlations (*p* < 0.05) were highlighted between the different quantities.

### 3.2. Classification Results

Results of the classification step are reported in Table 4 in terms of AUC and F1 score values, whereas results referring to the other metrics are included in Appendix C. Classification of the distances database was performed as a validation phase to assess the feasibility of classifying through the *FMI* database. The Random Forest algorithm showed the best score on the distances databases both in HC and PD cohorts, obtaining AUC values ranging between 94.3 and 91.6, and F1 scores between 76.2 and 71.5, respectively. By comparison, kNN was found to be the optimal technique in the classification with *FMI*; AUC values ranging between 88.9 and 88.4 and F1 scores between 70.1 and 73 were respectively obtained in the HC and PD datasets.

## 4. Discussion

Developing an automatic system for AU recognition is challenging due to the dynamic nature of facial expressions. Emotions are communicated by subtle changes in one or a few facial features occurring in the area of the lips, nose, chin, or eyebrows [30]. To capture these changes, different numbers of facial features have been previously proposed and, irrespective of their number, these landmarks cover the areas that carry the most important information, such as eyes, nose, and mouth [31]. Although more points provide richer information, they require more time to be detected. In order to quantify the involvement of each muscle with regard to each specific emotion, a face mobility index was developed based on distances between points of insertion of each muscle (see Figure 2 and Figure 3) coupled with significant facial features. A total index (*FMI*) was defined in order to summarize the overall face muscles involvement.

Based on these metrics, a population of PD subjects was compared with a group of healthy controls matched by age and gender. Through the distances analysis, a fine spatial characterization of movements related to muscle activity was obtained. Statistically significant differences were found among emotions between the two cohorts of subjects. According to [30], each emotion can be described by a specific set of AUs and this dataset highlighted impairments related to specific AUs and related muscles. A notable example of this involves the happiness emotion. Statistically significant differences were found in distances number 15, 32, 33, 34, and 35 in the lower part of the face; these quantities represent the movement of the combination of AUs 12 and 25, which are the characteristic AUs for happiness. Because AUs and face muscles are strictly related (see Appendix D), it can be noted that PD people displayed impairments in the Zygomatic Major and Depressor Labii muscles, and this finds agreement with [32]. Another example, considering the upper face, is the surprise emotion, described by AUs 1 and 2. Values greater than the neutral expression were found in both HC and PD people, but the latter displayed less mobility associated with those AUs corresponding to the Frontalis Muscle [33]. The anger and sadness emotions had statistically significant differences in the distances of the upper and lower face regions, respectively, showing deficits in the characteristic AUs 4 and 7 in anger, and AU 15 in sadness. It can be concluded that the corresponding muscles, Orbicularis Oculi and Triangularis, showed impairments in PD subjects. Fear displayed statistically significant differences in the upper region (distance number 36) associated with AUs 1 and 4 (Frontalis, Pars Medialis, and Corrugator Muscles), in the middle region associated with AU 20 (Risorius), and in the lower region associated with AU 25 (Orbicularis Oris). Finally, disgust revealed statistically significant differences in the upper region related to the activity of the Orbicularis Oculi muscle, and in the lower region in those distances associated with AU 17, in accordance with [34].

When considering face mobility in the overall metric, as expected, *FMI* reported general higher values in HC with respect to PD individuals even though only the happiness emotion revealed statistically significant differences. Whereas, when comparing the three *FMIs* in the upper, middle, and lower regions, it can be noted that happiness was still the most impaired in the middle and lower parts of the face. Furthermore, anger also showed statistically significant differences in the lower part between the two cohorts of subjects (Figure 6d), showing in PD people greater impairments in the related AU 24 and consequent Orbicularis Oris muscle.

Regarding the analysis of the correlation between the different demographic and clinical data, and the *FMI* values in the PD subjects, surprisingly, no significant correlations emerged. This may be interpreted as the ability of the proposed metric to measure different aspects of the symptom, which could be considered to be complementary to the standard clinical scales. In this regard, it is worth mentioning that UPDRS III primarily assesses patients’ appendicular function [35].

The classification algorithms showed good results in the preliminary analysis with the normalized distances databases. As expected, the AUC and F1 scores calculated on the HC individuals were higher than those of the PD cohort of subjects, despite the differences in the size of the datasets (17 vs. 50 subjects). These outcomes validated the possibility of using the new developed *FMI* index to perform classification and demonstrated the differences in expressivity in the two cohorts of subjects. The second step of classification involved the *FMI* datasets. Encouraging results were achieved even if performance values were inferior to those obtained with the former analysis. The kNN algorithm outperformed the other techniques in both HC and PD datasets.

Some limitations in the present study must be highlighted. Firstly, emotions were performed according to indications given by clinicians. This consideration can be overcome by naturally inducing the emotion by other stimuli (e.g., videos or movies); however, the downside of this approach is the uncertainty in the specific emotion that is elicited in the subject. Secondly, it is worth mentioning that the total UPDRS III score was employed in the correlation analysis. Furthermore, images were analyzed in the 2D image space, leading to a reduced accuracy in the measured quantities. The authors are aware of this limit, but this type of method was employed in order to simplify the setup, thus avoiding multiple camera acquisition and calibrations. In terms of classification, it is important to note that all the analyses were validated with the leave-one-out cross validation technique in order to cope with the limited sample of subjects. Finally, straightforward conventional machine learning techniques were employed rather than DL methods, which may be considered the most emerging approaches in this domain. However, due to its limited dataset, this study can be considered a feasibility analysis to assess whether this new index (*FMI*) may be an effective metric.

Future analysis could involve more advanced techniques, such as DL, increasing the number of subjects with relative *FMIs*. This approach will enable the introduction of automatic metric computation and real-time applications with possible time evolution analyses. Overall, the final aim of the proposed study could be the combination of all the proposed methods into a single easy-to-use tool to be adopted in clinical and research applications able to track disease progression, tailor targeted therapies, and evaluate their efficacy. Comparison among different rehabilitation interventions for hypomimia could be performed by assessing the new developed metric in the pre- and post-treatment conditions. Moreover, spontaneous emotion expressiveness could also be evaluated since this research includes emotions triggered by external instructions.

Nevertheless, other future investigations could be carried out in order to link the standard clinical assessment (UPDRS III items specifically related to hypomimia, i.e., facial expression and speech) with the proposed metrics.

Finally, by considering the relationship between face anatomical landmarks and muscle functions, future developments could also consider including the simultaneous acquisition of muscle activity through surface electromyography, as in [32,34], for validation purposes.

## 5. Conclusions

Although copious research has been undertaken on PD, hypomimia remains substantially under-investigated. The state-of-the-art research suggests evaluation of the symptom should be undertaken by means of clinical scales (UPDRS III item 3.2), which suffer from poor inter-rater repeatability, thus justifying the need to provide a more objective measure of facial expressiveness and recognition [5]. The present contribution showed the possibility of quantitatively characterizing the degree of hypomimia in the PD population. Moreover, through the proposed methodology, face muscles associated with a specific emotion (i.e., AU [9]) can be identified, thus providing a tool for planning target interventions. The overall metric represents a stand-alone methodology for measuring the degree of impairment without the need to be supported by the comparison with a database of healthy subjects [20]. Nevertheless, the application of the same methodology to the control group showed the ability to better highlight the specific impairment associated with PD, thus also supporting the adoption of such an index for classification purposes. Finally, both the proposed normalized distances and *FMI* can be considered a comprehensive description of face mobility that can become a powerful tool to quantitatively measure the degree of hypomimia associated with specific emotions in PD subjects.

## Figures and Tables

**Table 1:** Demographic data of participants (PD = subjects with Parkinson’s disease; HC = healthy control subjects).

	PD (*n* = 50)	HC (*n* = 17)
Gender	21 F	10 F
Age (years)	69.4 ± 7.79	66.56 ± 7.16
Disease duration (years)	8.28 ± 5.21	-
UPDRS (Part III)	36.19 ± 16.37	-

**Table 2:** Statistically significant ratios obtained per each emotion and face region. ns = non-significant.

Emotion	Upper Face	Middle Face	Lower Face
Fear	36	17, 19, 21, 22	39
Anger	2, 3, 4, 5	ns	ns
Disgust	2, 12	ns	35
Happiness	2, 3, 6, 7	14, 17, 20, 29	15, 32, 33, 34, 35
Sadness	ns	19, 24, 25	32, 34
Surprise	1, 2, 3, 4, 36	ns	9, 10, 11, 35

**Table 3:** Correlation coefficients between UPDRS III, duration, age, and gender values and *FMI* of each emotion. r = Pearson correlation coefficient, r_PB_ = Point-biserial correlation coefficient, *p* = *p* value (*p* < 0.05).

	(UPDRS III; *FMI*)		(Duration; *FMI*)		(Age; *FMI*)		(Gender; *FMI*)	
Emotion	r	*p*	r	*p*	r	*p*	r_PB_	*p*
Fear	0.012	0.933	0.144	0.335	0.038	0.796	0.155	0.278
Anger	0.015	0.920	0.114	0.447	0.085	0.555	0.031	0.827
Disgust	0.041	0.783	0.252	0.087	0.167	0.246	0.167	0.241
Happiness	0.005	0.970	0.070	0.639	0.068	0.640	0.030	0.833
Sadness	0.015	0.921	0.242	0.101	0.214	0.136	0.078	0.587
Surprise	0.099	0.503	0.017	0.909	0.212	0.139	0.080	0.578

**Table 4:** Results of supervised classification. AUC and F1 values are reported for the different classification methods. Dist_HC and Dist_PD refer to the dataset of normalized distances with the preprocessing step of PCA, whereas *FMI_HC* and *FMI_PD* refer to the *FMI* dataset.

	AUC [%]				F1 Score [%]			
	Dist_HC	Dist_PD	*FMI_HC*	*FMI_PD*	Dist_HC	Dist_PD	*FMI_HC*	*FMI_PD*
kNN	91.4 (±7.11)	89.4 (±4.03)	88.9 (±6.18)	88.4 (±3.75)	75.7 (±15.2)	71.2 (±5.77)	70.1 (±13.2)	73.0 (±1.7)
Tree	83.6 (±6.29)	83.3 (±5.80)	77.5 (±10.83)	82.0 (±3.10)	61.6 (±18.9)	67.6 (±7.84)	40.1 (±28.5)	59.2 (±8.7)
Random Forest	94.3 (±9.37)	91.6 (±3.39)	81.6 (±8.88)	82.5 (±4.94)	76.2 (±10.1)	71.5 (±5.88)	46.2 (±18.7)	62.9 (±6.9)
Neural Network	90.8 (±6.40)	89.0 (±5.79)	42.4 (±10.40)	60.5 (±10.95)	71.0 (±14.4)	63.5 (±12.8)	21.0 (±13.2)	25.1 (±23.9)
Naive Bayes	87.2 (±8.60)	80.7 (±7.01)	44.2 (±11.84)	52.0 (±10.86)	61.7 (±12.5)	43.4 (±19.6)	15.9 (±13.2)	30.3 (±11.1)
CN2 rule inducer	85.1 (±9.55)	85.0 (±3.86)	66.9 (±8.07)	71.4 (±5.58)	69.5 (±6.1)	73.4 (±4.89)	30.8 (±21.8)	44.5 (±12.8)

**Figure A1:**
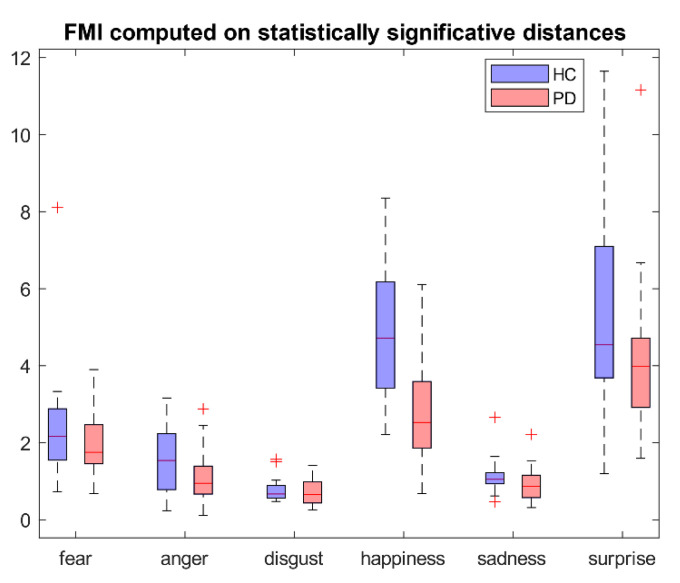
*FMI* computed on statistically significant different distances.

**Figure A2:**
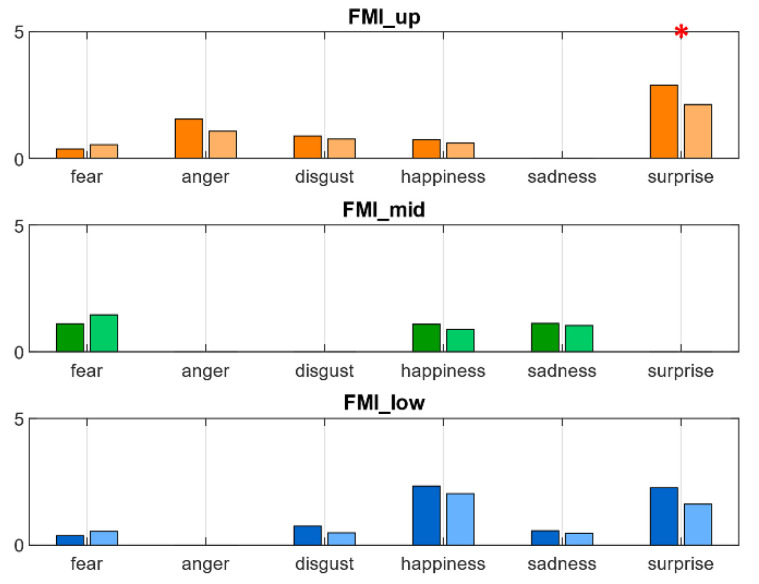
*FMI* computed on statistically significant different distances per face region. Red * highlights statistically significant differences at the 0.05 confidence level.

## Data Availability

The data presented in this study are available on request from the corresponding author. The data are not publicly available due to privacy.

## Abbreviations

The following abbreviations are used in the manuscript:

PD	Parkinson’s Disease
*FMI*	Face Mobility Index
FACS	Facial Action Coding System
AU	Action Unit
FER	Facial Expression Recognition
DL	Deep learning
HC	Healthy Controls
UPDRS	Unified Parkinson’s Disease Rate Scale
DSM	Diagnostic and Statistical Manual of Mental Disorders
MMSE	Mini Mental State Evaluation
kNN	k-Nearest Neighbors
PCA	Principal Components Analysis
AUC	Area Under the ROC Curve
ns	Non-Significant

## Appendix A

Power analysis for sample size estimation was applied according to [23] following equation (5) for unequal sized group. Chosen values of p value and power were *p* = 0.05 and power = 80% respectively. Values of *FMI* metric for happiness emotion were used from [24].

First, N was computed assuming that the groups were equal sized according to [23] Equation (2):

$$N=\frac{2}{d^{2}}\;c_{p,power}$$

where *N* is the required number of subjects in each group, d is the standardized difference (target difference/standard difference) and $c_{p,power}$ is a constant defined by the values chosen for the *p* value and power. In this case $c_{p,power}=7.9$ and according to [24]:

$$mean\;FMI\;HC=14.458;\;mean\;FMI\;PD=11.295;\;SD=3.5\;d=\frac{14.458-11.295}{3.5}=0.9037$$

Then *N* is adjusted according to the actual ratio of the two groups (*k*) with the revised total sample size *N*′:

$$N\prime =\frac{N{\left(1+k\right)}^{2}}{4k}$$

In this case *k* = 50/17=2.94.

Finally, the two individual sample sizes in each of the two groups are: *N*′/(1 + *k*) and *kN*′/(1 + *k*) resulting in:

$$N_{HC}=38.12\;subjects\;N_{PD}=12.96\;subjects$$

## Appendix B

*FMI* computed on statistically significant distances according to Equation (2) and distances presented in Table 2 is reported in Figure A1. No statistically significant differences were highlighted. Figure A2 represents *FMI* computed on the same quantities grouped by face regions. Statistically significant differences (*p* < 0.05) were found in surprise in *FMI_up*.

## Appendix C

Classification results for precision and recall metrics for the different classification techniques are reported in Table A1 and Table A2, respectively.

**Table A1:** Precision. Dist_HC and Dist_PD refer to the dataset of normalized distances with the preprocessing step of PCA whereas *FMI_HC* and *FMI_PD* refer to the *FMI* dataset.

	Precision			
	Dist_HC	Dist_PD	*FMI_HC*	*FMI_PD*
kNN	0.768	0.716	0.704	0.732
Tree	0.631	0.683	0.391	0.598
Random Forest	0.769	0.718	0.467	0.630
Neural Network	0.719	0.633	0.176	0.255
Naive Bayes	0.635	0.425	0.139	0.258
CN2 rule inducer	0.703	0.737	0.289	0.449

**Table A2:** Recall. Dist_HC and Dist_PD refer to the dataset of normalized distances with the preprocessing step of PCA whereas *FMI_HC* and *FMI_PD* refer to the *FMI* dataset.

	Recall			
	Dist_HC	Dist_PD	*FMI_HC*	*FMI_PD*
kNN	0.765	0.710	0.706	0.730
Tree	0.618	0.673	0.422	0.590
Random Forest	0.765	0.717	0.461	0.630
Neural Network	0.706	0.640	0.275	0.320
Naive Bayes	0.608	0.450	0.196	0.367
CN2 rule inducer	0.696	0.733	0.343	0.457

## Appendix D

Table A3 describs AUs, related names according to FACS [9] and corresponding muscles. Table A4 represents the basic emotions described by AUs according to [30].

**Table A3:** Description of the 44 AUs defined by Ekman and Friesen with their name and, when specified, corresponding muscles associated.

AU Number	FACS Name	Muscular Basis
1	Inner brow raiser	Frontalis, pars medialis
2	Outer brow raiser	Frontalis, pars lateralis
4	Brow lowerer	Depressor glabellae; depressor supercilli; corrugator
5	Upper lid raiser	Levator palpebrae superioris
6	Cheek raiser	Orbicularis oculi, pars orbitalis
7	Lid tightener	Orbicularis oculi, pars palebralis
9	Nose wrinkler	Levator labii superioris, alaeque nasi
10	Upper lid raiser	Levator labii superioris, caput infraorbitalis
11	Nasolabial fold deepener	Zygomatic minor
12	Lip corner puller	Zygomatic major
13	Cheek puffer	Caninus
14	Dimpler	Buccinator
15	Lip corner depresor	Triangularis
16	Lower lip depressor	Depressor labii
17	Chin raiser	Mentalis
18	Lip puckerer	Incisivii labii superioris; incisive labii inferioris
20	Lip stretcher	Risorius
22	Lip funneler	Orbicularis oris
23	Lip tightner	Orbicularis oris
24	Lip pressor	Orbicularis oris
25	Lips part	Depressor labii, or relaxation of mentalis or orbicularis oris
26	Jaw drop	Masetter; temporal and internal pterygoid relaxed
27	Mouth stretch	Pterygoids; digastric
28	Lip suck	Oribicularis oris
19	Tongue out	
21	Neck tightener	
29	Jaw thrust	
30	Jaw sideways	
31	Jaw clencher	
32	Lip bite	
33	Cheek blow	
34	Cheek puff	
35	Cheek suck	
36	Tongue bulge	
37	Lip wipe	
38	Nostril dilator	Nasalis, Pars Alaris
39	Nostril compressor	Nasalis, Pars Transversa and Depressor Septi Nasi
41	Lid droop	Relaxation of Levator Palpebrare Superioris
42	Slit	Orbicularis Oculi
43	Eyes closed	Relaxation of Levator Palpebrae Superioris
44	Squint	Orbicularis Oculi, Pars Palpebralis
45	Blink	Relaxation of Levator Palpebrae and Contraction of Orbicularis Oculi, Pars Palpebralis
46	Wink	Orbicularis Oculi

**Table A4:** Specific AUs involved in the basic emotions according to [30].

Emotion	AUs
Anger	4, 7, 24
Disgust	9, 10, 17
Fear	1, 4, 20, 25
Happiness	12, 25
Sadness	4, 15
Surprise	1, 2, 25, 26
